# IgG and IgA with Potential Microbial-Binding Activity Are Expressed by Normal Human Skin Epidermal Cells

**DOI:** 10.3390/ijms16022574

**Published:** 2015-01-23

**Authors:** Dongyang Jiang, Jing Ge, Qinyuan Liao, Junfan Ma, Yang Liu, Jing Huang, Chong Wang, Weiyan Xu, Jie Zheng, Wenwei Shao, Gregory Lee, Xiaoyan Qiu

**Affiliations:** 1 Department of Immunology, School of Basic Medical Sciences, Peking University, Beijing 100191, China; E-Mails: jiangdy@bjmu.edu.cn (D.J.); jing.ge.13@ucl.ac.uk (J.G.); lqy19870604@bjmu.edu.cn (Q.L.); majunfan@gmai.com (J.M.); liu_yang95@126.com (Y.L.); huangjing82@bjmu.edu.cn (J.H.); cwang@bjmu.edu.cn (C.W.); xuweiyanpzy@gmail.com (W.X.); shaoww23@bjmu.edu.cn (W.S.); 2 Center for Human Disease Genomics, Peking University, Beijing 100191, China; 3 Key Laboratory of Medical Immunology, Ministry of Health, Beijing 100191, China; 4 Hematology Oncology Center, Beijing Children’s Hospital, Beijing 100045, China; E-Mail: cutezjie@163.com; 5 Andrology Laboratory, University of British Columbia Center for Reproductive Health, Vancouver, BC V6H 3N1, Canada; E-Mail: leecyg@gmail.com

**Keywords:** immunoglobulin G, immunoglobulin A, human epidermis, VDJ rearrangement, microbial-binding

## Abstract

The innate immune system of the skin is thought to depend largely on a multi-layered mechanical barrier supplemented by epidermis-derived antimicrobial peptides. To date, there are no reports of antimicrobial antibody secretion by the epidermis. In this study, we report the expression of functional immunoglobulin G (IgG) and immunoglobulin A (IgA), previously thought to be only produced by B cells, in normal human epidermal cells and the human keratinocyte line HaCaT. While B cells express a fully diverse Ig, epidermal cell-expressed IgG or IgA showed one or two conservative V_H_DJ_H_ rearrangements in each individual. These unique VDJ rearrangements in epidermal cells were found neither in the B cell-derived Ig VDJ databases published by others nor in our positive controls. IgG and IgA from epidermal cells of the same individual had different VDJ rearrangement patterns. IgG was found primarily in prickle cells, and IgA was mainly detected in basal cells. Both epidermal cell-derived IgG and IgA showed potential antibody activity by binding pathogens like *Staphylococcus aureus*, the most common pathogenic skin bacteria, but the microbial-binding profile was different. Our data indicates that normal human epidermal cells spontaneously express IgG and IgA, and we speculate that these Igs participate in skin innate immunity.

## 1. Introduction

Immunoglobulin (Ig), also known as antibody, is thought to be produced exclusively by B lineage cells but no other cell types. B cell-expressed Ig is one of the key molecules for humoral immune responses; it recognizes foreign pathogens, neutralizes them directly, or activates other immune cells to remove them [1]. However, over the past 10 years, there is growing evidence that many normal or malignant non-B lineage cells, including certain gland epithelial cells [2,3,4,5,6,7], germ cells [8], endothelial cells [9], cardiomyocytes [10], or neurons [11,12], can also express Ig, including IgM, IgG [13,14] and IgA [3,15,16,17]. Both IgG and IgA derived from non-B cells promoted the survival and proliferation of cells [18,19,20,21,22]. Gland epithelial cell-derived IgM even exhibited natural antibody activity [6].

The skin is a barrier that limits the invasion and growth of pathogenic bacteria. Cutaneous antimicrobial defense mechanisms include the mechanical rigidity of the stratum corneum and its low moisture content, stratum corneum lipids, the production of lysozyme and antimicrobial peptides by the epidermis, acidity (pH 5), and defensins [23,24]. In addition, the skin supports the growth of commensal bacteria, such as *Staphylococcus epidermidis*, which protect the host from pathogenic bacteria, such as *Staphylococcus aureus*, both directly and indirectly [23,25]. There is, however, no direct evidence that Ig molecules can be expressed in the epidermis and contribute to antimicrobial activity. As long as 30 years ago, the cytoplasm of normal epidermal cells was reported to stain for IgG [26]. IgG and IgA deposits were frequently observed in epidermal cells and the epidermal stratum basale in several autoimmune diseases [27,28,29] and, occasionally, in normal skin [26,30]. In addition, several groups including us have reported that human malignant squamous cells, including esophageal, lung cancer and oral carcinoma, together with normal oral squamous cells adjacent to the tumors, frequently stained for IgG or IgA [5,19,31]. Based on these findings, we proposed that Ig molecules could be expressed in the epidermis and might provide antimicrobial activity.

Our study shows that normal skin epidermal cells stain significantly for IgG and IgA. The abundance of both IgG and IgA in the epidermis is verified by Western blot analysis and, importantly, we find evidence that the rearranged Igγ (IgG heavy chain) and Igα (IgA heavy chain) chains are transcribed in the epidermis. Unexpectedly, in each individual, the IgG and IgA heavy chain gene show unique VDJ rearrangements. Meanwhile, IgG and IgA protein and transcripts are also detected in HaCaT, an immortal human keratinocyte cell line. IgG and IgA derive from epidermis tissue or keratinocyte HaCaT could each recognize several pathogens, especially *Staphylococcus aureus*. These data suggest that normal human epidermal cells can express IgG and IgA, with unique VDJ rearrangements, and that this Ig could contribute to the innate immune of the skin.

## 2. Results and Discussion

### 2.1. Immunoglobulin G (IgG) and Immunoglobulin A (IgA) Staining in Normal Human Epidermis

**Figure 1:**
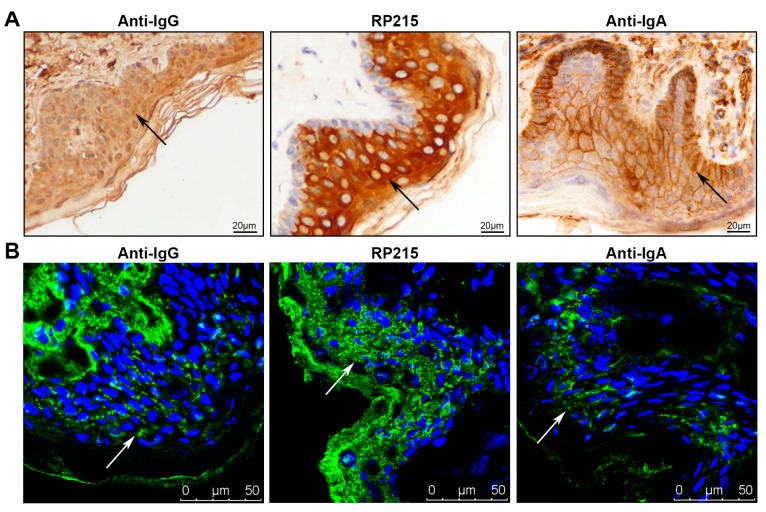
Immunoglobulin G (IgG) and immunoglobulin A (IgA) distribution in normal skin epidermis detected by immunostaining. (**A**) Immunohistochemical staining with commercial anti-IgG antibody, RP215 and anti-IgA antibody of paraffin-embedded sections of normal skin tissues. Arrows denote immunoreactivity of Ig; (**B**) Immunofluorescence staining with commercial anti-IgG antibody, RP215 and anti-IgA antibody in frozen sections of normal skin tissues. Green: immunostaining of Ig, blue: nucleus staining by Hochest 33342. Arrows denote immunoreactivity of Ig.

In this study, we used two anti-human IgG antibodies, the commercial anti-human IgG antibody and RP215, a monoclonal antibody that specifically recognizes a unique glycosylated epitope on IgG heavy chains that is over-expressed in certain non-B cancer cells but not in B cells [32,33]. As in our previous observations using the commercial anti-human IgG, both the epidermis and dermis stained, with the stronger staining in the dermis (Figure 1). Unexpectedly, RP215 stained the epidermis very strongly, but not the dermis. On the paraffin sections, we found strong positive staining by RP215 in cytoplasm of cells in the stratum spinosum and stratum granulosum, and weak positive staining in the stratum basale and stratum corneum (Figure 1A). On frozen sections by immunofluorescence, we found that RP215 strongly stained the stratum corneum and extracellular space, in addition to the cytoplasm of cells in the stratum spinosum and stratum granulosum (Figure 1B). The anti-human IgA showed obvious positive staining of the membranes and extracellular areas of epidermal cells, while their intracellular staining was weak. Exceptionally, basal cells stained strongly (Figure 1A). No immunoreactivity of anti-mouse or anti-rabbit IgG antibodies (as control) was observed in epidermal cells (Figure S1).

### 2.2. IgG and IgA in Epidermal Cells Confirmed by Western Blotting

To confirm the presence of IgG and IgA, we separated the epidermal layer from the foreskin samples by Dispase II digestion [34,35] and extracted the protein. Western blotting for the IgG heavy chains used commercial anti-human IgG antibody and RP215, and the IgA heavy chains used anti-human IgA antibody.

**Figure 2:**
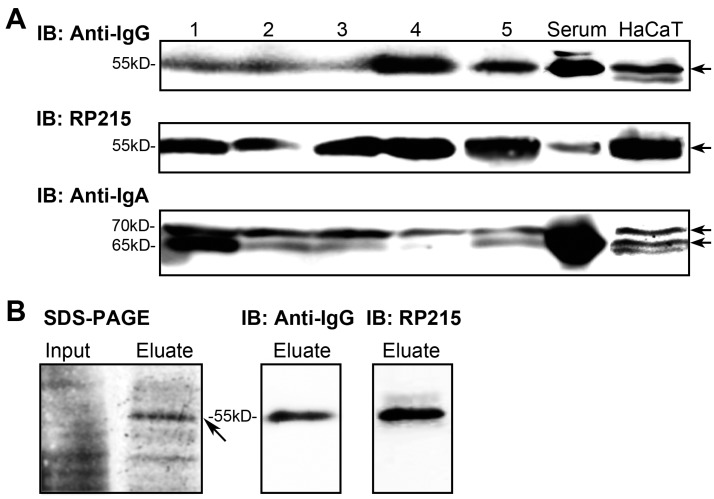
Expression of IgG and IgA in epidermal cells. (**A**) IgG or IgA expression in separated epidermal cells and HaCaT cell line determined by Western blotting using commercial anti-IgG antibody and RP215, or anti-IgA antibody, respectively. 1–5: epidermis samples. Arrows indicate immunoreactivity of IgG or IgA; (**B**) IgG purification by affinity chromatography with protein G sepharose. The protein band indicated by the arrow was further analyzed by mass spectrometry.

Clearly, both anti-human IgG antibody and RP215 recognized the same band at about *M*_r_ 55,000 in all five samples, which corresponds to the molecular size for IgG heavy chain (Figure 2A). As expected, RP215 recognized IgG heavy chains in epidermis, but not serum, more effectively than the commercial anti-human IgG. For further identification, we purified the IgG from the epidermis using protein G sepharose, which can bind most IgGs. The purified IgG in the eluate was recognized by both the commercial anti-human IgG antibody and RP215 (Figure 2B). Mass spectra results show that the band at *M*_r_ 55,000 bound by protein G contained segments of IgG heavy chain (Table S1).

The commercial anti-human IgA detected IgA heavy chain, at about *M*_r_ 65,000, in all five samples (Figure 2A). Interestingly, the epidermis contained the protein band at about *M*_r_ 70,000 that was also recognized by anti-IgA (Figure 2A). The change in molecular weight may result from a post-translational modification, such as glycosylation.

95% cells of epidermis are keratinocytes. To further confirm the endogenous production of IgG and IgA in epidermal cells, we examined IgG and IgA expression in HaCaT, a human immortalized keratinocyte cell line, by Western blotting. The immunoreactivity of IgG or IgA in HaCaT was similar with isolated epidermis, determined by commercial anti-human IgG antibody, RP215 and commercial anti-human IgA antibody (Figure 2A).

### 2.3. Rearranged IgG and IgA Heavy Chains Are Transcribed in Epidermal Cells

**Figure 3:**
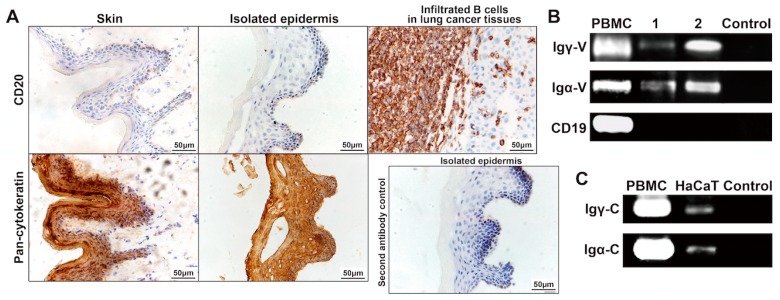
Transcription of IgG and IgA heavy chain gene in epidermal cells. (**A**) Immunohistochemistry staining of pan-cytokeratin (pan-CK) and CD20 in normal skin tissue and separated epidermis layer. Infiltrated B cells in the lung cancer tissue were used as positive control of CD20; (**B**) Rearranged IgG and IgA heavy chain variable region (Igγ-V and Igα-V) gene transcripts in isolated epidermis detected by RT-PCR. CD19 transcripts were detected to evaluate possible B cell contamination in isolated epidermis. PBMC: peripheral blood mononuclear cell as positive control; 1 and 2: epidermis samples from Case 1 and Case 2; control: PCR reaction mixture without templates as negative control; (**C**) IgG and IgA heavy chain constant region (Igγ-C and Igα-C) gene transcripts in HaCaT cell line detected by RT-PCR. PBMC as positive control and PCR reaction mixture without templates as negative control (control).

B lineage cells can produce abundant IgG and IgA. To test for possible contamination by B lymphocytes, we first examined the epidermal layer by immunohistochemistry for B lymphocytes, using anti-human CD20 antibody. The result clearly shows that no CD20-positive cells were found (Figure 3A). The isolated layers were proved only contained epidermis in which nearly all cells stained strongly for cytokeratin without dermis tissues through immunohistochemistry assay using anti-human pan-cytokeratin antibody (Figure 3A). Furthermore, RNA was extracted from the isolated epidermal layer and was reverse transcribed to cDNA. Contamination by B cells in the cDNA libraries was excluded by RT-PCR using the primers for amplifying CD19 gene, another specific marker for B cells. The cDNAs were tested for rearranged transcripts of IgG (γ) and IgA (α) heavy chains by RT-PCR using primers for the variable regions of the Ig heavy chain gene, and the respective constant region of Igγ or Igα chains. To detect Ig heavy chain gene rearrangements in the epidermis, we employed primers known to detect almost all V_H_DJ_H_ rearrangements in B cells [36]. Rearranged Igγ and Igα genes were successfully amplified from cDNA libraries of epidermis from two donors (Figure 3B). Furthermore, sequence analysis showed that all the epidermis-derived Igγ and Igα genes had classical VDJ rearrangements, with typical N nucleotide and P nucleotide additions at the V–D junctions and D–J junctions (Table S2) and that all rearrangements were functional, *i.e.*, that no stop codons were introduced by mutations or junctions between V, D and J segments. In addition, the constant region transcripts of Igγ and Igα were detected in HaCaT keratinocytes.

Surprisingly, however, the epidermal cell-derived V_H_DJ_H_ rearrangements showed only one or two VDJ patterns in each individual compared with the diverse B cell-derived V_H_DJ_H_ rearrangements observed in this study and elsewhere. The epidermis-derived Igγ gene showed only one VDJ pattern, VH1-2/DH5-18/JH6 in Case 1, and only one VDJ pattern, VH2-5/DH2-21/JH4 in Case 2. Epidermis-derived Igα genes showed two VDJ patterns, VH7-4-1/D6-13/JH6 and VH5-10-1/D2-2/JH3 in Case 1, and two VDJ patterns, VH1-3/D4-17/JH1 and VH1-3/D4-17/JH4 in Case 2, respectively (Table 1). None of the epidermis-derived VDJ rearrangement patterns of either Igγ or Igα chains was found in B cells in our study or in the NCBI database. Moreover, the epithelial Igγ VDJ rearrangements differed from any IgG heavy chain genes discovered in epithelial cancer cells [37]. The epidermis-derived Igγ and Igα sequences have been submitted to the GenBank database (GenBank Accession Numbers: JX420702–JX420707).

Generally, the B cell-derived V_H_DJ_H_ rearrangements of Igγ chains resembles that of Igα chains because both are derived from Igμ-expressing B cells, and class switch recombination changes only the constant region, from Igμ to Igγ or Igα. Interestingly, there was no identity between the V_Hγ_D_γ_J_Hγ_ and V_Hα_D_α_J_Hα_ rearrangements in the two cases studied (Table 1). This could suggest that epidermis-derived IgG and IgA might not arise by classical class switching.

**Table 1:** V_H_DJ_H_ recombinations of IgG and IgA heavy chain gene in epidermal cells.

Case		Igγ							Igα						
		Clone	No. of Clones		V_H_DJ_H_ Recombination		V Segment Identity (%) with Germlines		Clone	No. of Clones		V_H_DJ_H_ Recombination		V Segment Identity (%) with Germlines	
Case 1		1	1		VH1-2*02/D5-18*01/JH6*03		98.0		1	1		VH7-4-1*02/D6-13*01/JH6*02		84.0	
		2	1		VH1-2*02/D5-18*01/JH6*03		98.8		2	1		VH7-4-1*02/D6-13*01/JH6*02		84.3	
		3	1		VH1-2*02/D5-18*01/JH6*03		98.4		3	1		VH5-10-1*01/D2-2*01/JH3*03		79.9	
		4	1		VH1-2*02/D5-18*01/JH6*03		98.4		4	1		VH5-10-1*01/D2-2*01/JH3*03		81.3	
Case 2		1–6	6		VH2-5*02/D2-21*02/JH4*02		96.3		1–2	2		VH1-3*01/D4-17*01/JH1*01		77.4	
		7	1		VH2-5*02/D2-21*02/JH4*02		96.3		3	1		VH1-3*01/D4-17*01/JH1*01		78.8	
		8	1		VH2-5*02/D2-21*02/JH4*02		95.9		4	1		VH1-3*01/D4-17*01/JH1*01		79.2	
		9	1		VH2-5*02/D2-21*02/JH4*02		95.9		5	1		VH1-3*01/D4-17*01/JH4*02		79.2	
									6	1		VH1-3*01/D4-17*01/JH4*02		78.8	
PBMC (Control)		1	1		VH1-69*13/D2-21*01/JH4*02		80.6		1	1		VH1-46*01/D2-21*02/JH4*02		78.1	
		2	1		VH1-8*01/D6-13*01/JH4*02		83.0		2	1		VH1-2*02/D2-21*02/JH4*02		81.9	
		3	1		VH1-46*01/D1-7*01/JH4*02		90.5		3	1		VH1-3*01/D1-7*01/JH4*02		81.6	
		4	1		VH4-59*03/D6-6*01/JH6*02		94.8		4	1		VH4-59*08/D2-8*02/JH4*02		82.5	
		5	1		VH3-11*04/D5-12*01/JH5*02		85.7		5	1		VH1-18*01/D3-3*01/JH4*02		81.9	
		6	1		VH4-34*01/D3-10*01/JH6*02		92.9								
		7	1		VH3-23*01/D6-13*01/JH4*02		91.7								
		8	1		VH3-74*03/D1-26*01/JH5*02		91.8								

### 2.4. Epidermis-Derived IgG and IgA Recognized Foreign Pathogens

**Figure 4:**
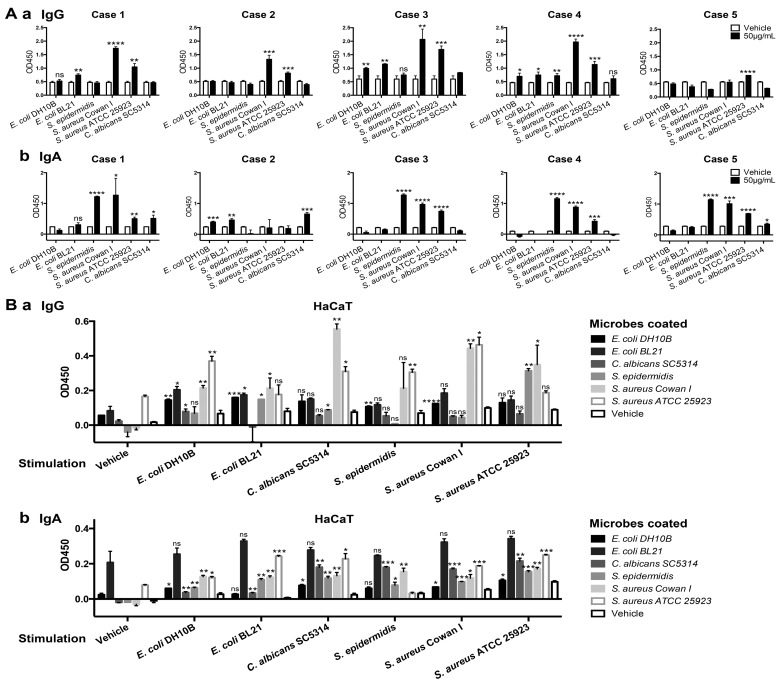
The binding of epidermis-derived IgG (**A**) and IgA (**B**) to *E. coli* strains DH10B and BL21, *S. aureus* strains Cowan I and ATCC 25923, clinical isolated strain of *S. epidermidis*, and *C. albicans* strain SC5314 was analyzed by ELISA. (**A**) Binding of IgG (**a**) and IgA (**b**) in epidermis tissue lysates from five healthy donors to microbes was analyzed by ELISA. 50μg/mL microbes were coated for testing Ig binding, vehicle as negative control. Data are presented as mean ± SD. Student’s *t*-tests were performed when mean of microbes-coated group was higher than vehicle control. Statistical significance: ns means no significant difference, * *p* < 0.05, ** *p* < 0.01, *** *p* < 0.001, **** *p* < 0.001 *vs.* vehicle control; (**B**) The microbial-binding activity of secreted IgG (**a**) and IgA (**b**) by HaCaT was analyzed by ELISA. Cell culture supernatant was collected 24 h after stimulation with different microbes (50 μg/mL). IgG and IgA with microbial-binding activity were captured from the supernatant by different microbes coated at 50 μg/mL and were detected with anti-IgG and anti-IgA antibodies, respectively. All data are calibrated by minus OD450 of culture medium control and presented as mean ± SD. Student’s *t*-tests were performed when mean of microbe stimulation group was higher than vehicle stimulation control. Statistical significance: ns means no significant difference, * *p* < 0.05, ** *p* < 0.01, *** *p* < 0.001, **** *p* < 0.001 *vs.* vehicle stimulation control.

As the first barrier of human body, the epidermis encounters and prevents various pathogens from entering, including bacteria and fungi. We tested the binding of IgG and IgA extracted from epidermis of five donors to *E. coli* strains DH10B and BL21, *S. aureus* strains Cowan I and ATCC 25923, a clinical isolate of *S. epidermidis*, and *Candida albicans* (*C. albicans*) strain SC5314, by ELISA. Of the five samples we examined, epidermis-derived Ig from most samples showed similar microbial-binding patterns. However, the reaction to different pathogens differed between IgG and IgA derived from epidermis. Epidermis-derived IgG could bind *S. aureus* strongly, as well as *E. coli*, and that epidermis-derived IgA mainly bound *S. epidermidis* and *S. aureus*, as well as *C. albicans* (Figure 4A). Furthermore, we detected the secretion of IgG and IgA in human keratinocyte cell line HaCaT by ELISA. Our results showed that microbial-binding IgG and IgA could be secreted to the culture supernatant, and that stimulation with microbes induced the secretion of either IgG or IgA with microbial-binding activity (Figure 4B). IgG spontaneously secreted by HaCaT could response to *S. aureus* ATCC 25923, and IgG binding to microbes, including *S. aureus* strains ATCC 25923 and Cowan I and *E. coli* DH10B, increased obviously after stimulation. Meanwhile, HaCaT-secreted IgA showed spontaneous anti-*E. coli* BL21 and *S. aureus* Cowan I activity, and after stimulation IgA binding ability to *S. aureus*, *S. epidermidis* and *C. albicans* increased obviously (Figure 4B). Furthermore, either HaCaT-secreted IgG or IgA stimulated by different microbe showed cross-recognition feature among different microbes. The microbial-binding profiles of HaCaT-secreted Ig were mostly consistent with epidermis tissue-derived Ig. These results suggest that epidermal cells could produce and secret Ig to recognize pathogens, and that epidermis-derived Ig is elevated after microbe stimulation and might participate in immune responses.

### 2.5. Discussion

Our study confirms our hypothesis that Ig molecules can be expressed in the epidermis, and they are involved in the innate immune of the skin.

Many reports previously showed that functional IgG and IgA, each with unique VDJ rearrangement patterns, were significantly expressed in squamous cell carcinoma cells [5,19,31]. Furthermore, IgG staining in normal epidermis has been reported [31], but it remains unclear whether normal squamous cells, such as the epidermis, can rearrange Ig genes and express functional Ig molecules. In this report, we first observed both IgG and IgA staining of normal human foreskin epidermal cells. Besides foreskin, non-prepuce normal skin tissues adjacent to melanoma were also detected by immunohistochemistry. Similar staining of IgG and IgA was observed using commercial anti-IgG, RP215 and anti-IgA (data not shown). However, due to the limit of obtaining absolutely “normal” non-prepuce skin tissues, we used foreskin for further investigation. The IgG staining was very strong in the normal skin epidermis, but not the dermis, when detected by RP215, a mAb which specifically recognizes a unique glycosylated epitope on IgG heavy chain that is over-expressed in some non-B cancer cells [32,33]. Western blotting confirmed that IgG heavy chain was recognized by both RP215 and commercial anti-IgG in epidermal cells. This finding suggests that the epidermis-derived IgG bears a unique glycosylated epitope on its heavy chain, which is different from conventional IgG but similar to non-B cancer cell-derived IgG, and which can be specifically recognized by RP215 instead of commercial anti-IgG antibody.

Ig gene rearrangement and transcription is a prerequisite for Ig expression. To exclude the possibility that extracellular Ig is transferred into epithelial cells through the Fcgamma receptor or the pIgR (polymeric immunoglobulin receptor) on the plasma membrane of epidermal cells [38,39], we looked for rearranged transcripts of IgG and IgA genes in the epidermal cells. We excluded possible contamination of the cDNA libraries by B lymphocytes and were still able to amplify functionally rearranged V region gene transcripts for IgG and IgA heavy chains from cDNA of isolated normal epidermis.

We compared the epidermis-derived VDJ rearrangements of IgG and IgA heavy chain transcripts of two individuals with B cell-derived Ig. The variable regions of epidermis-derived IgG and IgA displayed conventional VDJ rearrangements like B cell-derived Ig, such as typical N and P nucleotides at the junctions between V and D segments, and between D and J segments. However, there were still some differences between VDJ rearrangements in epidermal cells and those in B cells. First, such as other non-B cell-derived Ig, the epidermis-derived IgG and IgA heavy chains had limited rearrangement patterns in each individual, contrasting with the diversity of B cells, but the epidermis from the two individuals whose were tested had different VH (V region of Ig heavy chains) rearrangements. Second, the V segments used in the two samples were not those commonly used in B cells (the frequencies of these V segment in B cells are: VH1-2: 2.78%, VH2-5: 1.27%, VH7-4-1: 1.84%, VH5-10-1: 0.57%, and VH1-3: 0.76% [40]). Third, the epidermal cell-derived IgG and IgA, like Ig derived from epithelial cancer cells or CD33+ myeloblasts sorted from AML patients, seemed not to originate from classical class switching [37,41,42]. IgM-producing cells are precursors of the IgG and IgA producers in classical Ig class switching, so that the Igγ and Igα gene assembly modes should be similar. However, we did not detect any identical of V_Hγ_D_γ_J_Hγ_ and V_Hα_D_α_J_Hα_ patterns in the cases studied. This unexpected result suggested that Igγ chain was completely different from the Igα chain in the same epidermal cells and that IgG and IgA production in these cells did not follow the classical class switching mechanism.

The epidermis mainly consists of keratinocytes. Therefore, we used HaCaT, a keratinocyte cell line, to further confirm the expression of IgG and IgA by epidermal cells. Western blot assay revealed that keratinocyte cell line HaCaT could also produce IgG and IgA. Meanwhile, IgG and IgA heavy chains transcripts were detected in HaCaT cells.

Although the skin has a tight mechanical barrier, it may be breached by pathogens such as *Staph. aureus* and *Strep. pyogenes*. Previous reports indicated that human epithelial cells can spontaneously produce IgM with natural antibody activity by recognizing microbial antigens [6]. We therefore used *S. aureus*, *S. epidermidis*, *C. albicans* and *E. coli* to test the microbial-binding activity of epidermis-derived IgG or IgA of five individuals for these pathogenic or commensal bacteria. Epidermis-derived Ig from most samples could recognize certain pathogens and showed similar reaction to different pathogens. To further investigate the microbial-binding of epidermis-derived Ig without the potential contamination of B cell-derived Ig, we analyzed the secretion and microbe-recognizing activity of Ig in human keratinocyte cell line HaCaT. HaCaT could spontaneously secret IgG with anti-*S. aureus* activity and IgA with anti-*E. coli* and anti-*S. aureus* activity. However, after microbe stimulation, secreted Ig was obviously elevated and showed similar microbial-binding profiles with Ig extracted from epidermis tissues. Thus, we suggest that microbial-recognizing antibody production by the epidermis constitutes a novel cutaneous defense mechanism that protects from bacterial infection. Interestingly, IgG and IgA secreted by HaCaT stimulated by different microbes showed cross-recognition feature among different microbes. The detailed mechanism needs further investigation. Moreover, epidermis-derived IgG and IgA displayed different microbial-binding patterns. Epidermis-derived IgG bind strongly to *S. aureus*, the leading cause of skin infections, while epidermis-derived IgA could bind both *S. aureus* and the commensal bacterium *S. epidermidis* well. The potential correlation between the different localization or limited VDJ recombination patterns of IgG and IgA and their different microbial-binding patterns is still to be discussed.

## 3. Experimental Section

### 3.1. Tissue Samples, Bacterial Strains and Cell Line

Fresh foreskin tissue samples from normal adults were obtained from Peking University Third Hospital. Human peripheral blood from healthy donors was provided by Beijing Red Cross Blood Center. One lung cancer tissue samples with infiltrated B lymphocytes, as the positive control tissue of CD20 immunostaining, was obtained from Peking University Third Hospital.

*Escherichia coli* (*E. coli*) strains DH10B and BL21 (Biomed, Beijing, China), *Staphylococcus aureus* (*S. aureus*) strains Cowan I (Sigma, St. Louis, MO, USA) and ATCC 25923, *Staphylococcus epidermidis* (*S. epidermidis*) clinical isolate strain (donated by Hui Wang, Peking University People’s Hospital, Beijing, China), and *Candida albicans* SC5314 (donated by Ruoyu Li, Peking University First Hospital, Beijing, China) were used in this study.

Written informed consent was obtained from all donors. All procedures were performed with the ethical approval of the Clinical Research Ethics Committee of the Peking University Health Science Center.

### 3.2. Cell Culture

The human immortalized keratinocyte cell line HaCaT was a gift from Xuejun Zhu (Peking University First Hospital, Beijing, China). HaCaT cells were cultured in Dulbecco’s modified Eagle’s medium (Invitrogen, Carlsbad, CA, USA) supplemented with 10% fetal bovine serum (FBS; Hyclone/Thermo Fisher Scientific Inc., Waltham, MA, USA).

### 3.3. Immunohistochemistry

Human tissues were fixed in 10% formalin, embedded in paraffin, and sectioned. After deparaffinization, antigen retrieval was conducted in Tris–EDTA buffer (pH 9.0) boiled at 120 °C for 2 min with autoclave. The slides were incubated with 3% hydrogen peroxide for 5 min, and blocked in 10% normal goat serum for 10 min. After removing excess blocking buffer, the slides were incubated with RP215 (gift of Gregory Lee), rabbit anti-human IgG antibody (1:3000; Dako, Glostrup, Denmark), rabbit anti-human IgA antibody (1:150; Dako), rabbit anti-human pan-cytokeratin antibody (1:50; Santa Cruz, Santa Cruz, CA, USA), or rabbit anti-CD20 antibody (Epitomics, Burlingame, CA, USA) as the primary antibody, at 37 °C for 1 h, rinsed, and incubated with horseradish peroxidase-conjugated (HRP) goat anti-mouse/rabbit IgG antibody (1:100; Dako) as the secondary antibody at 37 °C for 30 min. The bound antibodies were detected using DAB (Dako).

### 3.4. Immunofluorescence Staining and Confocal Microscopy Analysis

For frozen sections, the fresh foreskin tissues were frozen directly in liquid nitrogen, embedded in Tissue-Tek OCT compound (Sakura, San Marcos, CA, USA), sectioned and fixed with acetone. The frozen sections were blocked in 5% BSA for 30 min and incubated with primary antibodies RP215, rabbit anti-human IgG antibody (1:500; Dako), or rabbit anti-human IgA antibody (1:100; Dako) at room temperature for 1 h, followed by incubation with Dylight 488-conjugated rabbit anti-mouse or goat anti-rabbit IgG antibodies (1:400; Jackson, West Grove, PA, USA) at room temperature for 30 min. Nuclei were stained with Hochest 33342 (5 μg/mL; Sigma) for 5 min at room temperature. Images were captured by confocal microscopy using a Leica TCS SP5 imager (Leica Microsystems, Mannheim, Germany).

### 3.5. Isolation of Epidermis

Epidermal sheets were dissociated as previously described [34]. Briefly, following incubation with Dispase II (2.4 U/mL; Roche, Rotkreuz, Switzerland) in DMEM overnight at 4 °C, the epidermal sheet was mechanically separated from the dermis and used for protein extraction or RNA isolation.

### 3.6. Isolation of Mononuclear Cells from Peripheral Blood

Peripheral blood was obtained from healthy donors. Mononuclear cells were isolated from peripheral blood using Ficoll/Hypaque (Second Chemistry Factory, Shanghai, China) density-gradient centrifugation, as previously described [37]. Peripheral blood mononuclear cells (PBMCs) at the interface were recovered and washed with PBS, and the isolated PBMCs were used for total RNA extraction.

### 3.7. Protein Extraction and Purification

Separated epidermis was treated with RIPA lysis buffer (1% Triton X-100, 0.1% sodium dodecyl sulfate, 1% sodium deoxycholate, 0.15 M NaCl and 10 mM Tris (pH 7.2)) with protease inhibitor cocktail (Roche) on ice for 30 min. After centrifugation for 15 min at 12,000× *g* at 4 °C, the lysate supernatant was collected for Western blotting, enzyme-linked immunosorbent assay, or IgG purification by affinity chromatography as follows. The lysate was added to protein G sepharose from which, after washing in PBS, the bound IgG was eluted by 0.1 M Glycine-HCl (pH 2.4). The fractions collected were analyzed by SDS-PAGE, Western blotting and mass spectrometry (performed by the Beijing Genomics Institute). HaCaT cells were lysed using TSD lysis buffer (1% sodium dodecyl sulfate, 5 mM dithiothreitol and 50 mM Tris (pH7.5)) with protease inhibitor cocktail (Roche) at room temperature for 20 min. After centrifugation for 15 min at 12,000× *g* at 4 °C, the lysate supernatant was collected for Western blotting.

### 3.8. SDS-PAGE and Western Blotting

Briefly, protein samples were analyzed in 12.5% SDS-PAGE and stained with Coomassie Brilliant Blue. For Western blot analysis, the proteins were separated by electrophoresis and transferred from the gel to nitrocellulose membranes, where they were stained indirectly with RP215, goat anti-human IgG antibody (Sigma), or rabbit anti-human IgA antibody (Dako), the primary antibodies listed above, and HRP-conjugated donkey anti-mouse or anti-rabbit IgG antibodies (Jackson) as secondary antibodies. Bound secondary antibodies were visualized by electrochemiluminescence (Thermo, Waltham, MA, USA).

### 3.9. RT-PCR

RNA was extracted from HaCaT cells, isolated epidermis or PBMCs following the standard RNeasy Mini Kit (Qiagen, Hilden, Germany) protocol. Reverse transcription was carried out with oligo (dT) primer using Sensiscript RT Kit (Qiagen) according to the manufacturer’s instructions. Semi-nested touchdown PCR was then performed to amplify variable region transcripts of IgG and IgA heavy chain, using Phusion DNA Polymerase (Thermo), at annealing temperatures from 60 to 56 °C for 35 cycles, with sense primers located in variable region (VH1, VH2, VH3, VH4, VH5 and VH6) in both PCR reactions, coupled with antisense primers located in CH1 domain of IgG (CγCH1) or IgA (CαCH1) in the first round of PCR and antisense primer in J region (JH) in the second round of PCR. The constant region transcripts of IgG and IgA were amplified at the annealing temperature of 55 °C for 35 cycles. To exclude B lymphocyte contamination of the skin cDNA libraries, CD19, a B lymphocyte marker, was amplified with the annealing temperature of 50 °C for 35 cycles. Human PBMCs were used as positive controls of Ig and CD19 amplification. Primers used in this study were listed in Table S3. PCR products were cloned into pGEM-T Easy Vector (Promega, Madison, WI, USA) and sequenced by ABI 3730XL Genetic Analyzer (Applied Biosystem, Foster City, CA, USA).

### 3.10. Sequence Analysis

All sequences were submitted to the IMGT V-QUEST program to analyze the usage and junctions of Ig heavy chain variable region genes. Alignments with published sequences were by BLAST tool in NCBI (the National Center for Biotechnology Information), and alignments among sequences used Lasergene software (DNAStar, Madison, WI, USA).

### 3.11. Microbes Stimulation and Enzyme-Linked Immunosorbent Assay

To test whether epidermis-derived IgG and IgA could recognize pathogens, we used enzyme-linked immunosorbent assays (ELISA). Microtiter plates were coated with formaldehyde-fixed *E. coli* strains DH10B or BL21, *S. aureus* strains Cowan I or ATCC 25923, clinical isolates of *S. epidermis*, or *Candida albicans* strain SC5314, diluted in carbonate-bicarbonate coating buffer (pH 9.6) at 50 μg/mL at 4 °C overnight. After blocking the plates with 3% BSA for 1 h at 37 °C, the lysate from individual isolated epidermal tissues were added to the wells (50 μg/well), and the plates were incubated for 2 h at 37 °C. Rabbit anti-human IgG or IgA antibodies and HRP-conjugated goat anti-rabbit IgG antibody were used to detect IgG or IgA, with tetramethylbenzidine as the substrate. OD450 was measured using a microplate reader (Bio-Tek, Winooski, VT, USA).

For further exploration, we stimulated HaCaT with different pathogens and detected the microbial-binding activity of secreted IgG and IgA in supernatant by ELISA. HaCaT was seeded in the 6-well plate. After 12 h, the six formaldehyde-fixed pathogens mentioned above were respectively added to the culture medium at 50 μg/mL, vehicle as control. The culture supernatants were collected after another 24 h. ELISA assays were conducted as mentioned above, except that the lysate from epidermal tissues were replaced with the culture supernatants at 50 μL/well.

### 3.12. Statistical Analysis

All statistical analyses were performed using GraphPad Prism 6.0 software (Graphpad, San Diego, CA, USA). Differences between groups were calculated by Student’s *t*-test and were considered to be statistically significant when *p* < 0.05.

## 4. Conclusions

This study illustrated for the first time that normal human epidermal cells, including normal epidermal cells from foreskin tissues and keratinocyte line HaCaT, could spontaneously express functional IgG and IgA. In epidermis, IgG was found primarily in prickle cells, and IgA was mainly detected in basal cells. In contrast to B cell-derived Ig displaying diversity, epidermal cell-expressed IgG or IgA showed one or two conservative V_H_DJ_H_ rearrangements in each individual. Moreover, IgG and IgA from epidermis of the same sample exhibited different VDJ rearrangement patterns. Furthermore, both epidermis-derived IgG and IgA showed potential antibody activity by binding pathogens such as *Staphylococcus aureus*. However, more studies *in vitro* are needed to investigate the role of epidermis-derived IgG and IgA in skin local immunity and the detailed mechanism.

Our data indicate that normal human epidermal cells spontaneously express IgG and IgA epidermis-derived IgG and IgA, and we speculate that these Igs participate in skin innate immunity.

## Supplementary Materials

Supplementary materials can be found at  http://www.mdpi.com/1422-0067/16/02/2574/s1.
